# Systems Behavior: Of Male Courtship, the Nervous System and Beyond in *Drosophila*

**DOI:** 10.2174/138920208786847980

**Published:** 2008-12

**Authors:** B Dauwalder

**Affiliations:** Department of Biology and Biochemistry, University of Houston, Houston, TX 77204-5001, USA

**Keywords:** Courtship, *fruitless*, *doublesex*, fat body, *takeout*, sex determination, *Drosophila.*

## Abstract

Male courtship in fruit flies is regulated by the same major regulatory genes that also determine general sexual differentiation of the animal. Elaborate genetics has given us insight into the roles of these master genes. These findings have suggested two separate and independent pathways for the regulation of sexual behavior and other aspects of sexual differentiation. Only recently have molecular studies started to look at the downstream effector genes and how they might control sex-specific behavior. These studies have confirmed the essential role of the previously identified male specific products of the *fruitless* gene in the neuronal circuits in which it is expressed. But there is increasing evidence that a number of non-neuronal tissues and pathways play a pivotal role in modulating this circuit and assuring efficient courtship.

## INTRODUCTION

One of the fascinating fields of neurogenetics is the study of complex behaviors and the genes that control them. Courtship behavior in fruit flies (*Drosophila melanogaster*) is particularly well suited for such studies. The behavior consists of a series of consecutive steps that the courting male performs and that can easily be observed and quantified (reviewed in [1,2]): The male orients himself toward the female, taps the female with his forelegs, extends and vibrates one wing to “sing” a courtship song, licks the female’s genitalia, attempts copulation, and copulates. Thanks to the many genetic and molecular tools that exist for this model organism it has been possible to gain significant insight into the genes and processes that regulate the behavior.

**Fig. (1):**
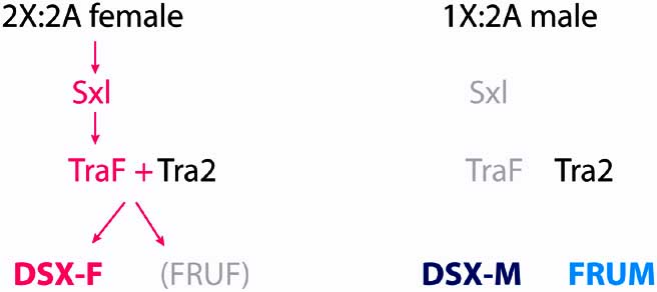
Simplified version of the *Drosophila* sex determination pathway.

In *Drosophila melanogaster* development, sex is determined cell- autonomously or by signals between adjacent tissues and not by hormones (reviewed by [3,4]). Sexual behavior in flies is regulated by the same master regulators that control general somatic sexual development and are part of a cascade of alternative splicing events. The primary signal lies in the ratio of X-chromosomes to autosomes, which determines whether a functional form of the “master regulator” protein *Sex-lethal* (*Sxl*) is produced (in females) or not (in males) (Fig. **1**). In females, functional *Sxl* protein acts as a splicing regulator to control female-specific expression of the transformer (TraF) protein, itself a splicing regulator. TraF interacts with Tra-2, another splicing regulator. Together they control the female-specific splicing of *doublesex* (*dsx*) and *fruitless* (*fru*) pre-mRNAs. This results in the production of the female-specific *dsx* protein (DSX-F). No female specific FRU protein is formed because of translational control [5,6]. In males, the absence of TraF leads to the default splicing of both *dsx* and *fru* RNAs and to the production of male-specific dsx (DSX-M) and FRU^M^ proteins. The central role of *tra* in the control of sexual differentiation and sex specific behavior is demonstrated by the fact that chromosomal females with a mutation in the *tra* gene are transformed into normal males with male courtship behavior [7]. 

Since *tra* controls both *dsx* and *fru*, further studies have examined which one of them controls mating behavior by examining the courtship behavior of *dsx* and *fru* mutant males. A mutation in *dsx* was found to reduce overall male courtship and to impair courtship song, but it did not abolish courtship [8,9]. Females that expressed male DSX-M acquired male morphology, but did not court [8]. In contrast, males with strong mutant alleles of *fru* barely courted, demonstrating that *fru* is essential for male courtship. Weaker *fru* mutations lowered courtship and caused males to indiscriminately court females and males [10-14]. Based on these and similar experiments it was proposed that there are two independent branches downstream of *tra*, one through *dsx* that controls somatic sexual differentiation outside the nervous system, and another through *fru* in the nervous system that controls male courtship behavior [15]. In recent years it has become increasingly evident, however, that the two pathways are both significantly contributing and interacting to regulate male courtship through both the CNS and other tissues. This article will review the role of *fru* in regulating courtship and discuss recent evidence that there is a close interplay between *dsx* and *fru* regulated pathways and genes in the regulation of courtship.

## FRU IS A MASTER REGULATOR OF MALE SPECIFIC BEHAVIOR

Recent excellent reviews have described the complexities of the *fru* gene and its functions in detail [16-18]. This article will summarize some of this information and focus on more recent findings on how FRU^M^ may control an amazing array of behaviors. A central role for *fru* in male courtship has recently been confirmed by findings that FRU^M^ and the neuronal network defined by FRU expressing neurons are sufficient to specify the early steps of male mating behavior [19,20]. Expression of FRU^M^ in otherwise completely normal females leads to male courtship behavior towards other females, although at lower levels than in control males and with impaired courtship song, indicating that factors other than FRU^M^ are also required. In addition, females that express FRU^M^ show male specific aggression, another sex specific behavior that is regulated by *fru* [21-23]. 

The fruitless gene is large (150 kb) and encodes numerous transcripts with non-sex-specific and sex-specific functions that are transcribed from several promoters [6,11-13,24]. The most distal promoter, P1, gives rise to the sex specific transcripts. They contain TraF binding sequences in their second exon. Binding of TraF, which is only present in females, leads to the choice of an alternative 5’ splice site and inclusion of sequences with numerous translational stop codons. This female specific transcript appears to be unable to produce any protein [6]. In males, in the absence of TraF binding, the stop codon containing part of the transcript is spliced out, thus allowing a long uninterrupted reading frame that gives rise to the male specific FRU^M^ protein [6,11-13,25]. Fru proteins belong to the BTB-Zn-finger protein family, suggesting that they act as transcription factors, although no direct molecular targets have been identified yet. However, genome-wide searches for genes that are controlled by *fru* have identified numerous target genes (see later). The male specific FRU^M^ protein contains a unique 101 amino acid N-terminal region. These sequences are highly conserved among *Drosophila* species. Their male specific function is still under investigation. A recent report has suggested that these residues are essential to allow FRU^M^ to function when it is ectopically expressed in otherwise normal females, but that they may be less important for FRU^M^ function in its normal male context [26]. It has already been demonstrated that FRU^M^ isoforms that contain one of several alternative putative DNA binding domains affect male neuronal differentiation and behavior differently [27].

The FRU^M^ protein is expressed in about 2000 neurons of the brain and ventral ganglia, as well as in the peripheral nervous system [5,11-13,19,20,28,29]. Are these neurons unique to males, and is this how FRU exerts its functions? That this is not the case was recently shown by the generation of transgenic flies that contained a manipulated *fru* gene that exclusively spliced the sex specific transcript in a male mode, even in females. To visualize the protein made from this transcript, FRU coding sequences were replaced with sequences coding for the yeast transcription factor Gal4 whose expression can be visualized, thus marking cells that usually express the male specific splice form. When this transgene was expressed in females, the expression pattern was basically indistinguishable from the pattern normally seen in males, indicating that the neuronal circuits that express FRU^M^ in males are present in females [19,20,28]. Therefore, there are no gross anatomical differences caused by FRU^M^ expression that can account for *fru* dependent male behaviors. However, on a smaller scale, neuronal dimorphism may be part of *fru* regulation. There are several FRU^M^ expressing clusters that differ in males and females by cell number and other characteristics. And there is increasing evidence for specific roles for these and other subsets of FRU^M^ expressing cells. A cluster of FRU^M^ expressing neurons that are part of the median bundle, a structure that receives sensory input, is involved in controlling the sequential order of the different courtship steps, perhaps by coordinating different sensory stimuli [30]. Two glomeruli in the antennal lobe which receive olfactory input (DA1 and VA1v) differ in size between males and females, and those two glomeruli, plus an additional one (VL2A), were found to be the only olfactory glomeruli that were innervated by fru-Gal4 positive neurons [20,31]. Olfactory neurons that project to the DA1 glomerulus express the Or67d olfactory receptor which responds to 11-cis-vaccenyl acetate, a male derived pheromone (cVA). Activation of the receptor with cVA has different functions in males and in females: In males, it inhibits courtship to other males, in females it acts to stimulate receptivity towards males [32,33]. Recent experiments have shown how the same pheromone perceived by the same receptor might lead to different behaviors in males and females. The projections from the DA1 glomerulus to the protocerebrum, a higher order brain center, were found to be sexually dimorphic. The male specific projection pattern is dependent on the expression of FRU^M^ in these neurons and other FRU^M^ positive cells [34]. In yet another cluster in the brain, named fru-mAL, neuron number and morphology is different between males and females. These differences depend on FRU^M^ and its regulation of differential programmed cell death between males and females [29]. Intriguingly, this cluster of neurons has recently been implicated in the control of male specific aggressive behavior [35].

These data demonstrate that *fru* expressing clusters can have distinct male specific functions. It is not known how this functional specificity is brought about. Part of the specificity might be due to the fact that these clusters are part of different and dedicated neuronal circuits. Since FRU^M^ has the characteristics of a transcription factor it is likely to bestow male specific molecular characteristics to the neurons that express it. This could occur both during the development of these neurons and/or by setting differential physiological states of individual neurons in the adult animal. Whether the same set of FRU^M^-dependent transcripts is induced in all *fru* expressing neurons or whether subsets of *fru* clusters express specific signatures of *fru* regulated genes remains to be seen.

## BOTH FRU^M^ AND DSX ARE REQUIRED IN THE CNS FOR MALE SPECIFIC FUNCTIONS

The male courtship song is an important part of male courtship behavior that has been shown to map to certain regions of the brain and the ventral thoracic ganglia [36]. *fru* mutant males have impaired courtship song, indicating a role for *fru* in regulating the behavior [13,14]. FRU^M^ however does not appear to be sufficient for specifying normal courtship song, since females expressing FRU^M^ do not exhibit normal courtship song [37]. Since a mutation in *dsx* also causes impaired courtship song in males, Rideout *et al.* and others tested the possibility that both *fru* and *dsx* are required to specify normal male courtship song [8,37]. Indeed, expression of both FRU^M^ and the male form of DSX, DSX-M, was required for normal courtship song. Co-expression of FRU^M^ and DSX-M was observed in neurons of the mesothoracic ganglia in a neuronal cluster that shows a sexually dimorphic number of FRU^M^ expressing neurons [37]. Intriguingly, expression of DSX-M was required to obtain the full set of male FRU^M^ expressing neurons. This is reminiscent of previous findings that both DSX-M and FRU^M^ are required in the abdominal ganglia for the differentiation of male-specific serotonergic neurons [27], and that DSX-M is required for an increased number of neurons in the abdominal ganglia of males [38]. A central role for FRU^M^ expressing abdominal neurons in the production/performance of courtship song was shown recently by Clyne *et al*. [39]. The authors used a light-activated ion-channel that they expressed in all *fru*-expressing neurons. This allowed them to specifically activate these neurons by light. When the cells in the abdomen of decapitated flies were activated, both males and females extended a wing and performed courtship song, although the characteristics of the song were different in females. When the females also expressed FRU^M^, the displayed song was very male-like and was recognized by control females as valid courtship song. The authors concluded that the potential to display the behavior was largely present in both sexes, but whether it was initiated, and the quality of the song was dependent on stimuli and/or coordination mediated by FRU^M^. In contrast to the results obtained in decapitated flies, light activation of the behavior occurred at very low frequency in intact flies. Since control of courtship song does not only require male abdominal ganglia, but also male posterior regions of the brain, it is possible that the light-activated response was suppressed in intact flies, because sensory stimuli that usually trigger the behavior were absent and higher-order control neurons were therefore inhibiting the display of the behavior.

That females may possess some intrinsic neural pathways for courtship has previously been suggested by findings that females which lack FRU^M^, but are mutant for the gene *retained (retn)*, show some male courtship [40]. *retn* codes for a ARID-box transcription factor that is expressed in a small subset of neurons in both males and females that does not overlap with *fru* expressing neurons. Furthermore, the effect of *retn* is influenced by whether DSX-M or DSX-F is present in these flies and the authors showed that *fru* and *dsx* can act together in the context of developmental genes such as *retn*. 

DSX-M was also found to control the expression of a male-specific gustatory receptor, Gr68a. It is expressed in taste sensillae on the male foreleg and may play a role in the pheromonal perception of females. Removal of Gr68a by RNAi affects courtship [41].

## THE FAT BODY, A NON-NEURONAL TISSUE, AND GENES EXPRESSED OUTSIDE THE *FRU* CIRCUITS ARE REQUIRED FOR NORMAL COURTSHIP

Both FRU^M^ and DSX are transcription factors, but very little is known about the sex-specific genes they regulate and what role they might play in courtship. Their identification is crucial for our understanding of courtship regulation. Several groups have performed molecular screens to identify sex specific transcripts and transcripts that change in *fru* and *dsx* mutants [41-45]. However, the biological role of only a few of these transcripts has been examined so far. The *takeout* (*to*) gene was identified in a subtractive screen and was shown to be preferentially expressed in male heads [44]. A mutation in *takeout* affects male courtship behavior and interacts genetically with *fru*, indicating that they act in the same overall pathway that regulates mating behavior. *takeout* mutant males showed an overall reduction in courtship; although they were able to perform all steps of courtship, they initiated and maintained the behavior at a significantly lower rate. Given that the mutant affects mating behavior, it was surprising when it was found that the *takeout* transcripts were not present in the nervous system, but that the gene was male-specifically expressed in the fat body that surrounds the brain (there is also some non-sex specific expression in the antennae, the olfactory organs of the fly) [44]. The insect fat body consist of large, lipid-filled cells and is often compared to the mammalian liver (Fig. **3**). Its crucial role in fat storage, energy metabolism and immunity is well documented [46-48], but it had not been implicated in the control of sex specific behaviors before. Its only known sex specific role was in the production of yolk proteins in females [49]. To test whether there is a general sex specific role for the fat body in male courtship behavior, genetic means were used to feminize the fat body in otherwise normal males and to ask whether this affected courtship. To do so, the female specific TraF protein was targeted only to fat body cells. To change sex only in a defined subset of cells is feasible in flies because, as mentioned earlier, sex is determined cell-autonomously and not regulated by circulating hormones. Courtship was reduced drastically in males with feminized fat body, indicating that the sexual identity of the fat body is indeed crucial for normal courtship [50]. Interestingly, courtship in these males was considerably lower than in the *takeout* mutants, suggesting that the feminization did not just reduce the amount of *takeout*, but probably also that of other fat body transcripts which normally play a role in courtship regulation. These other transcripts remain to be identified. The lower courtship scores observed in males with feminized fat body are reminiscent of the reduced scores observed in females that express FRU^M^. What if courtship in FRU^M^ females was lower than normal because they still had a female fat body? In a genetic experiment that did the opposite of the one just described in males, the fat body tissue was masculinized in females that also express FRU^M^. These females now courted as well as normal males, underscoring the importance of the fat body and its interaction with the CNS [50]. There is increasing evidence for a sex specific role of fat body factors. In another screen for sex specifically expressed transcripts in the head, Fuji *et al*. [41] identified four genes with preferential sex specific expression in the fat body. *tsx*, *sxe1*, *sxe2 *were male-specifically, and *fit* was female specifically expressed. In addition, recent genomic screens have identified a number of sex-specifically expressed genes that appear to be expressed in the fat body [45], see below.

How can a tissue like the fat body regulate courtship behavior? As discussed earlier, expression of FRU^M^ in the CNS is required to establish the competence for courtship behavior. Obviously, fat body factors need to interact with the nervous system to regulate its function. Since the fat body is a major secretory tissue, one possibility is that it does so by secreting factors into the hemolymph, the circulating fluid of flies, and that these factors somehow interact with the brain. Consistent with this hypothesis is the finding that the Takeout protein is present in the hemolymph [50]. This suggests that soluble, circulating factors may play a significant role in the control of *Drosophila* sexual behavior, reminiscent of the hormonal control of behavior in vertebrates (Fig. **3**). How such proteins cross through or signal through the blood brain barrier and interact with *fru* circuits is unknown. Two lines of evidence suggest that the sex-specific role of fat body factors is physiological, in the adult and behaving fly, rather than during development: Only feminization of fat body in adult flies, but not at larval stages, leads to the described reduction in courtship [50]. And, in experiments that looked at transcriptional changes in adult males that were allowed to court females for 5 minutes, at least three out of eleven up-regulated genes were genes that are controlled by the sex determination pathway and are expressed in the fat body [51].

**Fig. (2). takeout expression requires both doublesex and fruitless.**
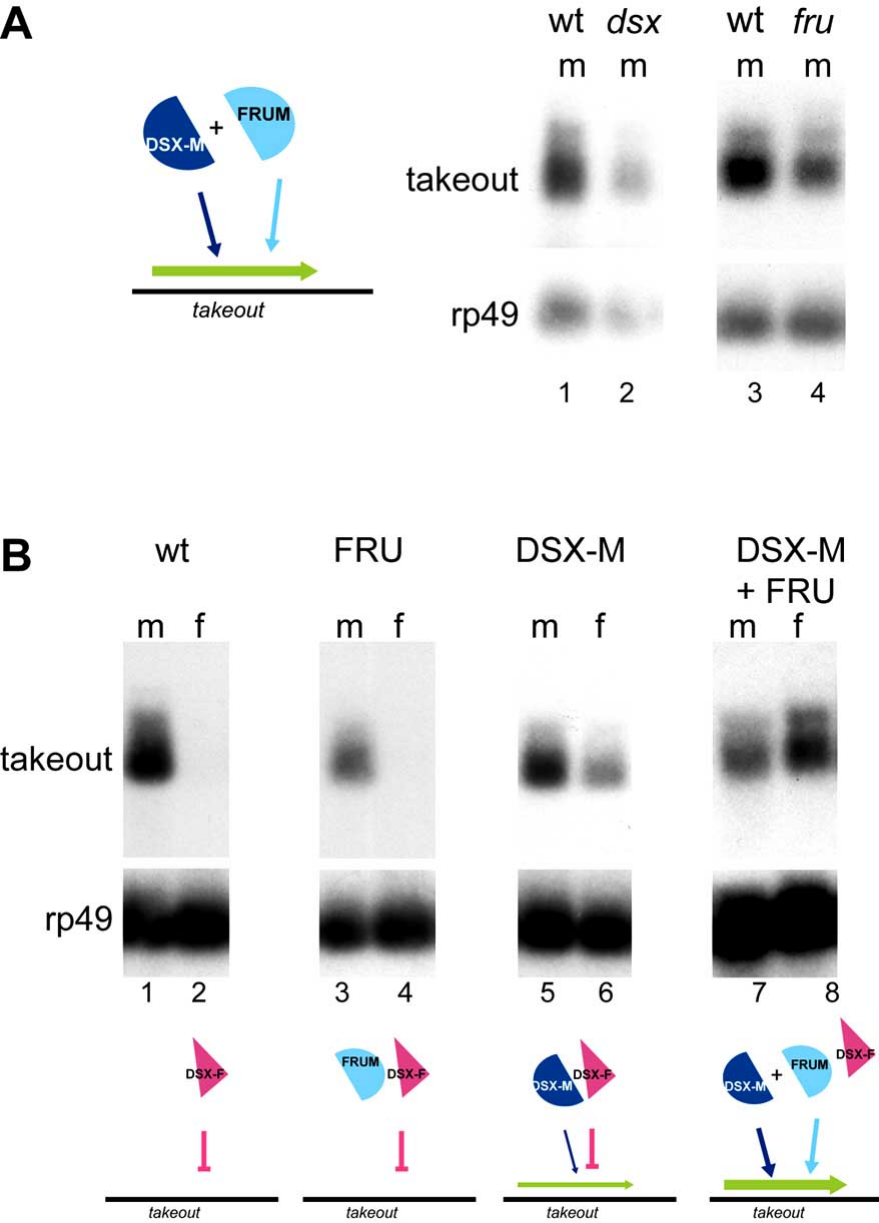
(**A, B**) Northern analysis of *takeout* expression and cartoon interpretation of *takeout* transcriptional regulation in males (**A**) and females (**B**). Note that while both DSX-M and FRU^M^ are required there is no evidence that they bind directly or interact physically with each other. (**A**) *takeout* expression in *dsx^1^* (lane 2) and *fru^4^/fru^3^* mutant males is reduced (lane 4), when compared to their heterozygous siblings (lanes 1 and 3). (**B**) Forced expression of male-specific forms of *dsx* and *fru* act synergistically to induce *takeout* in XX individuals. Females expressing the male DSX-M from the dominant mutation *dsx^SWE^* show activation of *takeout* (lane 6) compared to control females (lane 2). These females are *dsx^SWE^/ dsx^+^* and produce both *dsx^F^* and *dsx^M^*. There is no effect of *dsx^SWE^/ dsx^+^* on *takeout* expression in males (compare lanes 5 and 1). No significant induction of *takeout* was observed in chromosomal females expressing *fru* in fat body (lane 4). Females expressing both *fru* and *dsx^SWE^* (lane 8) have *takeout* expression levels indistinguishable from their male siblings (lane 7). *takeout* expression in wildtype males and females is shown in lane 1 and 2 (Modified with permission from Dauwalder *et al.* Genes & Dev., Nov 2002; 16: 2879 – 2892, Copyright 2002, Cold Spring Harbor Laboratory Press).

**Fig. (3). Sex specific factors from the fat body are essential for courtship.**
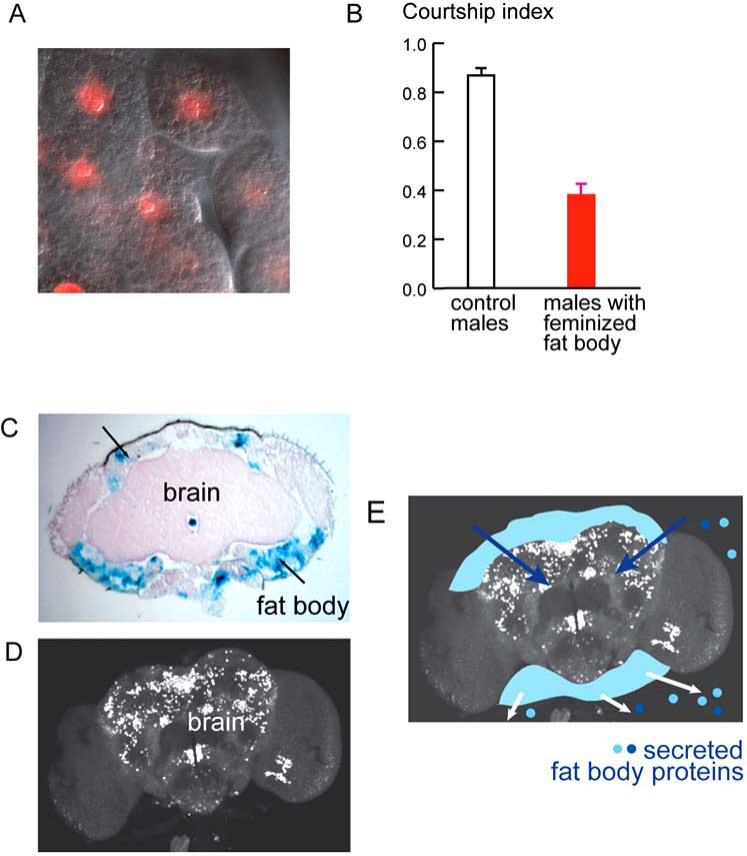
(**A**) Fat body cells are large and contain lipid droplets (for visualization, the nuclei were marked with a fluorescent protein (dsRed Stinger). (**B**) Courtship performance of wildtype males and males with feminized fat body. The courtship index is the fraction of time a male courts within the 10 minute observation period. n=10, error bars indicate s.e.m. (**C**) Frontal section of a fly head. Fat body cells are marked by X-Gal staining (blue, arrows). The fat body surrounds the brain. (**D**) Isolated whole brain showing *fru* expressing cells (picture courtesy of Dornan *et al.* 2005). (**E**) Model for fat body – brain interaction. It is proposed that the fat body secretes factors that enter the hemolymph, the circulating fluid, reach the brain and interact with *fru*-expressing neuronal circuits (Modified with permission from Dauwalder *et al.* Genes & Dev., Nov 2002; 16: 2879-2892, Copyright 2002, Cold Spring Harbor Laboratory Press, Lazareva *et al.* PLoS Genet 3(1): e16. doi:10.1371/journal.pgen.0030016) and Dornan *et al.* Genesis 2005; 42:236–246, copyright 2005, Wiley-Liss, Inc. Reprinted with permission of Wiley-Liss, Inc., a subsidiary of John Wiley & Sons, Inc).

The *takeout* gene codes for a 27kD protein with characteristics of soluble carrier proteins that is most similar in sequence to secreted Juvenile Hormone binding proteins of other insects [44,52,53]. Interestingly, expression of the *takeout* gene is regulated by both DSX-M and FRU. Mutations in either gene reduce the amount of *takeout* present in males, indicating that both *fru* and *dsx* are required for full *takeout* activation [44] (Fig. **2**). These findings support the notion that DSX-M acts as an activator in males, as had been previously suggested [54-56]. The action of DSX proteins is best characterized in the case of the female specific yolk protein (*yp2*) promoter. Both DSX-F and DSX-M bind the *yp2* promoter; bound DSX-F activates transcription, whereas bound DSX-M represses its activation [56-58]. Thus, the described effects of DSX proteins on *yp2* are opposite to those observed for *takeout* regulation. In contrast to the regulation observed in *yp2*, however, both DSX-M and FRU^M^ are required for normal *takeout* expression. Consistent with this, expression of FRU^M^ in females alone is not sufficient for male levels of *takeout*, most likely due to the absence of DSX-M and an inhibitory effect by the presence of DSX-F (Fig. **2**). Only in females that express both FRU^M^ and DSX-M are wildtype levels of *takeout* expression observed. It is not known yet whether DSX and FRU act by directly binding to the *takeout* promoter, or through other transcription factors. Potential DSX consensus binding sites [59] have been observed within 1kb upstream of the takeout transcription start site. FRU recognition and binding sequences have not been described yet.

Recent micro-array based genomic studies that examined the expression of genes that are regulated by the sex determination hierarchy in the heads of flies have identified new modes of DSX regulated gene expression [45]. These studies suggest that the model of regulation that is seen in yolk protein genes and *takeout*, namely that one form activates and the other represses, is true only for a subset of *dsx* regulated transcripts. For others, expression was lower in both sexes when *dsx* was mutated, indicating that they are usually activated by both DSX-M and DSX-F. Another class was higher in both mutants, indicating that both DSX forms usually repress these transcripts. The reason why these genes were found to be expressed at different levels in the two sexes in the first place was that DSX-F appeared to both activate and repress to a greater extent. This may be due to the fact that DSX-F interacts with another protein encoded by *intersex *[60], which could make it a more potent activator and repressor. In addition, there was a class of transcripts where DSX was only required in one sex. The same study also identified genes that were regulated by FRU^M^. When whole heads and dissected brains were compared, it was discovered that a majority of the identified DSX and FRU^M^ targets was expressed outside of the nervous system. These genes are most likely expressed in the fat body, or perhaps in glial cells. These findings indicate that there may be a fairly large number of sex specific transcripts in the fat body, supporting earlier findings about its sex specific function. Further studies will be required to determine the role of individual genes and whether/how they contribute to sex specific behaviors. 

Since FRU^M^ expression has so far not been observed in fat body [5,6,11], the finding that a significant number of its transcripts are regulated by *fru* poses the question of how this regulation occurs. Unless *fru* levels in the fat body were below detection threshold, FRU^M^ probably acts indirectly, perhaps by influencing the generation of a circulating signal, or *via* other effects mediated by neuronal activity of FRU^M^ expressing cells. Very few FRU^M^ targets were identified in the nervous system, possibly because they are expressed only in small subsets of FRU^M^ expressing cells and therefore may not have been detected under the stringent criteria of the screen [45]. One of the identified FRU^M^ targets, *dpr* (*defective proboscis extension response*), was found to affect courtship. Mutant males showed reduced courtship latency and reduced time to copulation. Interestingly, *dpr* was expressed in ascending median bundle neurons that express FRU^M^ and in earlier studies had been shown to regulate the timing of courtship [30].

Not only is there mounting evidence for the crucial role of the fat body, but in addition, a recent study by Grosjean *et al*. [61] has shown a contribution of glial cells in the brain. A mutation in the gene “*genderblind*” which is expressed in CNS glial cells, causes males to become non-discriminatory and court females and males alike. This is most likely due to their overreaction to and improper processing of chemosensory cues, since they do not court *desat1* mutant males which produce very small amounts of sex specific pheromones. However, they do court *desat1* males that have been “painted” with 7-tricosene, a pheromone that is thought to normally prevent male-male courtship. *genderblind* codes for a transporter that regulates extracellular glutamate, an indication that glutamatergic neurons are involved in the processing of pheromone detection.

Taken together our current knowledge of male courtship behavior shows an intricate network of neuronal circuits that are set up under the control of both the *fruitless* and *doublesex* genes and that together confer the neuronal competence for the behavior (also discussed in [62]). However, perhaps surprisingly, efficient and normal courtship is dependent on additional input from non-neuronal tissues, such as the fat body and glial cells. Diffusible sex specific factors secreted from the fat body may play an important role in this regulation, suggesting that sexually dimorphic characters in *Drosophila* result from the interaction of sex-determining genes and endocrine factors.
